# Diabetic nephropathy: the role of inflammation in fibroblast activation and kidney fibrosis

**DOI:** 10.3389/fendo.2013.00007

**Published:** 2013-02-06

**Authors:** Keizo Kanasaki, Gangadhar Taduri, Daisuke Koya

**Affiliations:** ^1^Division of Diabetology and Endocrinology, Kanazawa Medical UniversityKahoku, Japan; ^2^Department of Nephrology, Nizam’s Institute of Medical SciencesHyderabad, India

**Keywords:** fibroblasts, kidney fibrosis, EMT, EndMT, cytokines, diabetic nephropathies, inflammation

## Abstract

Kidney disease associated with diabetes mellitus is a major health problem worldwide. Although established therapeutic strategies, such as appropriate blood glucose control, blood pressure control with renin–angiotensin system blockade, and lipid lowering with statins, are used to treat diabetes, the contribution of diabetic end-stage kidney disease to the total number of cases requiring hemodialysis has increased tremendously in the past two decades. Once renal function starts declining, it can result in a higher frequency of renal and extra-renal events, including cardiovascular events. Therefore, slowing renal function decline is one of the main areas of focus in diabetic nephropathy research, and novel strategies are urgently needed to prevent diabetic kidney disease progression. Regardless of the type of injury and etiology, kidney fibrosis is the commonly the final outcome of progressive kidney diseases, and it results in significant destruction of normal kidney structure and accompanying functional deterioration. Kidney fibrosis is caused by prolonged injury and dysregulation of the normal wound-healing process in association with excess extracellular matrix deposition. Kidney fibroblasts play an important role in the fibrotic process, but the origin of the fibroblasts remains elusive. In addition to the activation of residential fibroblasts, other important sources of fibroblasts have been proposed, such as pericytes, fibrocytes, and fibroblasts originating from epithelial-to-mesenchymal and endothelial-to-mesenchymal transition. Inflammatory cells and cytokines play a vital role In the process of fibroblast activation. In this review, we will analyze the contribution of inflammation to the process of tissue fibrosis, the type of fibroblast activation and the therapeutic strategies targeting the inflammatory pathways in an effort to slow the progression of diabetic kidney disease.

## INTRODUCTION

Diabetic nephropathy is the most common cause of end-stage renal disease (ESRD) worldwide (Ritz et al., 1999; Viswanathan, 1999; Parving, 2001; Remuzzi et al., 2002). Current treatment strategies may slow but in most of cases cannot arrest progression toward ESRD (Lewis et al., 1993; Brenner et al., 2001). Once diabetic nephropathy progresses to ESRD, management with dialysis is expensive and is associated with increased cardiovascular morbidity and mortality compared to non-diabetic ESRD (Parving, 2001; Remuzzi et al., 2002).

Therefore, in an effort to aid in the discovery of novel therapeutic concepts that allow the prevention and retardation of diabetic nephropathy, innovative insight into the pathophysiology of diabetic nephropathy is mandatory (Wolf and Ritz, 2003). Hemodynamic alterations such as hyperfiltration and hyperperfusion caused by hyperglycemia are considered major kidney injury factors, but such alterations are only one aspect of a complex series of pathophysiological alterations related to the presence of glucose metabolism defects.

Various theories have been proposed concerning the pathogenesis of diabetic nephropathy, including proteinuria, genetics, race, hypoxia, ischemia, and inflammation (Fernández Fernández et al., 2012). Individually, the theories proposed thus far may not be able to explain the progression of diabetic nephropathy. Among these theories, inflammation appears to be the critical pathway for the development and progression of diabetic nephropathy.

The role of inflammation in diabetic nephropathy has been reported previously (Mezzano et al., 2003; Rivero et al., 2009; Wada and Makino, 2013). In the context of current theories that are focused on inflammation and fibroblast activation, such as epithelial-to-mesenchymal transition (EMT) and endothelial-to-mesenchymal transition (EndMT), we discuss the role of inflammation in the progression of diabetic kidney disease, with an emphasis on therapeutic strategies.

## GENERAL OVERVIEW OF THE BIOLOGY OF DIABETIC NEPHROPATHY

### MULTIPLE PROFIBROTIC PATHWAY INVOLVEMENT

Various theories have been proposed regarding the development and progression of diabetic kidney disease. The pathological events that have traditionally been implicated in the development of diabetic nephropathy include poor glycemic control, hypertension, proteinuria, and other such factors. Individual factors such as genetics, race, obesity, and smoking have been shown to contribute to variations in the onset and progression of kidney disease (Bakris et al., 2003; American Diabetes Association, 2011; Cravedi et al., 2012).

Diabetic organ damage, including diabetic nephropathy, is fundamentally triggered by glucose metabolism defects; normalizing blood glucose levels is essential for diabetic therapy (The Diabetes Control and Complications Trial Research Group, 1993; Ohkubo et al., 1995; Tu et al., 2010). Indeed, pancreatic transplantation in overt diabetic nephropathy with sclerotic glomeruli can cure the nephropathy with significant amelioration of renal pathology, but the process takes nearly 10 years (Fioretto et al., 1993, 1998; Fioretto and Mauer, 2011). However, the normalization of blood glucose levels in diabetic patients is challenging, and such intensive therapies in diabetic patients are associated with increased mortality risk and are likely associated with frequent severe hypoglycemia (Ismail-Beigi et al., 2010). Although strict control of blood glucose levels inhibits the progression of urine albuminuria levels, it does not offer any difference in clinical kidney disease outcome (Hemmingsen et al., 2011; Coca et al., 2012; Slinin et al., 2012). Therefore, to prevent diabetic complications, therapeutic strategies are required in addition to those that target blood glucose normalization.

The degree of urine albumin/protein is associated with the progression of kidney disease via the activation of tubular angiotensin-converting enzyme (ACE) inhibitors or inflammatory pathways (Abbate et al., 2006; Cravedi et al., 2012; Erkan, 2012). Strategies to decrease proteinuria with renin–angiotensin system (RAS) blockade have been shown to be partially renoprotective (Lewis et al., 1993; Brenner et al., 2001). However, 30% of diabetic nephropathies occur in the absence of significant proteinuria (Fernández Fernández et al., 2012), suggesting that other pathways have a role in the pathogenesis of the condition, and recent clinical trials raise questions about the significance of strong RAS blockade using a combination of RAS inhibitors in diabetic nephropathy patients (Mann et al., 2008; Parving et al., 2012).

Systemic/intra-glomerular hypertension can result in the onset and progression of diabetic kidney disease. ACE inhibitors and angiotensin receptor blockers (ARBs) have been shown to slow disease progression by controlling hypertension, in addition to decreasing proteinuria (Lewis et al., 1993; Brenner et al., 2001). Non-dihydropyridine calcium channel blockers, aldosterone antagonists, and direct rennin inhibitors (e.g., aliskiren) have been reported to control hypertension and reduce albuminuria/proteinuria, but evidence of long-term, significant clinical renal outcomes remains lacking (Slinin et al., 2012).

Diabetic insults to the kidney are mediated through the various pathogenic pathways described above. Inflammation appears to be the final common pathway and can result in either kidney repair if regulated or fibrosis if the process is uncontrolled.

### INFLAMMATION AND KIDNEY FIBROSIS

Fibrosis is a sequence of normal wound healing and repair that is activated in response to injury to maintain the original tissue architecture and ensure normal functional integrity (Pinzani, 2008; Wynn, 2008). Essentially, inflammation is required for tissue repair, except in embryos, where tissue can be repaired without typical inflammation (Bullard et al., 2003; Redd et al., 2004). Inflammation is closely associated with tissue repair, regeneration of parenchymal cells, and filling of tissue defects with fibrous tissue, i.e., scar formation (Wynn, 2007). The inflammatory response to an injury eventually results in either (a) the normal tissue repair process and the regaining of structural and functional integrity or (b) abnormal and uncontrolled tissue repair, which leads to progressive fibrosis with loss of tissue structure and function. Although non-resolving inflammation has been shown to be a major driving force in the development of fibrotic disease, as described above, inflammation is an essential aspect of host defense mechanisms in response to injury (Wynn, 2007). In this regard, progressive fibrosis with sustained inflammation can be considered as a type of chronic wound with defects in the normal healing process (Liu, 2011). Thus, controlling excessive inflammation has great therapeutic potential of inhibiting progressive kidney fibrosis.

Once tissue is injured, inflammatory cells infiltrate the site of injury due to the enrichment of pro-inflammatory niches at the site of injury and directional guidance mediated by chemotactic cytokine concentration gradients (Chung and Lan, 2011). The infiltration of inflammatory cells, such as lymphocytes, monocytes/macrophages, dendritic cells (DCs) and mast cells, precedes the process of tissue fibrosis (Chung and Lan, 2011). At the injury site, activated infiltrated inflammatory cells can synthesize tissue damage factors such as reactive oxygen species (ROS) and produce fibrogenic cytokines and several growth factors (Ricardo et al., 2008; Duffield, 2010; Vernon et al., 2010). Sustained inflammatory cell activation results in profibrotic cytokine pressure within the local microenvironment. The cytokine pressure subsequently primes the fibroblasts at the site of injury and induces tubular epithelial cell phenotypic changes into a mesenchymal-like phenotype that produces a large amount of profibrotic extracellular matrix (ECM) components (Liu, 2011). Therefore, sustained inflammation after the tissue injury could be the initiator, the trigger and the activator of tissue fibrosis progression.

The importance of inflammation in the development and progression of renal fibrosis has been well documented (**Table 1**; Ricardo et al., 2008; Duffield, 2010; Vernon et al., 2010). Inflammation is regulated by the complex interaction of various factors, involving cytokines, chemokines, and adhesion molecules. Renal inflammation is characterized by glomerular and tubulointerstitial infiltration by inflammatory cells, including neutrophils, macrophages, lymphocytes, and other such cells, regardless of the initial injury. Such cellular infiltrates are evident in both experimental models of renal disease and human renal biopsy specimens (Ferenbach et al., 2007). Inflammation is initiated with the entry of neutrophils, which take up cell debris and phagocytose apoptotic bodies (Lee and Kalluri, 2010). Activated neutrophils degranulate to release inflammatory and profibrotic cytokines (Lee and Kalluri, 2010). Subsequently, macrophages infiltrate damaged tissues and play an important role in the production of inflammatory cytokines and profibrotic cytokines (Lee and Kalluri, 2010). The recruitment and activation of T lymphocytes has been shown to be a significant early event in the initiation of renal fibrosis, and it typically precedes the influx of macrophages into the injured kidneys (Lin et al., 2008). The importance of T or B lymphocytes has been analyzed in genetic mouse models that lack mature B and T lymphocytes and have a V(D)J recombination-activating protein 1 (RAG1) deficiency. RAG1-deficient mice are protected against fibrosis after obstructive injury (Tapmeier et al., 2010).

**Table 1:** Inflammatory cell types and their roles in kidney fibrosis.

Inflammatory cell type	Major roles in the kidney fibrosis process	Reference
Neutrophil	Initiation of inflammation Uptake of cell debris Phagocytosis of apoptotic bodies	Lee and Kalluri (2010)
Lymphocyte	T cell recruitment and activation as an early event in the fibrosis process T cell deficiency associated with reduced fibrosis Cytokine production	Lin et al. (2008), Tapmeier et al. (2010)
Macrophage	M1 macrophages exhibit a pro-inflammatory phenotype, and M2 macrophages do not have a typical inflammatory phenotype Generation of various cytokines, chemokines and reactive oxygen species	Ricardo et al. (2008), Lin et al. (2009), Duffield (2010), Wang and Harris (2011)
Dendritic cell	Capture and deliver antigens to T cells Stage-specific role in kidney fibrosis	Heymann et al. (2009), Macconi et al. (2009), Hochheiser et al. (2011a)
Mast cell	Controversial role in fibrosis Mast cell-deficient mice display increased morality and kidney fibrosis in experimental animal models	Miyazawa et al. (2004), Kanamaru et al. (2006), Timoshanko et al. (2006),Holdsworth and Summers (2008)

Similar anti-fibrogenic effects were observed when CD4^+^ T cells were depleted in wild-type mice after obstructive injury (Tapmeier et al., 2010), whereas reconstitution with purified CD4^+^ T cells in RAG1-knockout (B, T cell-deficient) mice led to restored fibrogenic responses following obstructive injury (Tapmeier et al., 2010), suggesting that lymphocytes, especially CD4^+^ T cells, have a critical role in the pathogenesis of renal fibrosis induced by obstructive injury. An analysis of type IV collagen α3 chain-deficient mice, the model of human Alport syndrome, revealed that RAG1 deficiency in mice significantly ameliorated tubulointerstitial injury without amelioration in glomerular basement membrane (GBM) structures (LeBleu et al., 2008), but streptozotocin (STZ)-induced diabetic animal models using the same RAG1-deficient mice displayed no alteration in tubular injury when compared to control diabetic mice, even though RAG1-deficient diabetic mice exhibited low levels of albuminuria (Lim et al., 2010). Macrophage infiltration into the kidney cortex was the same in the STZ-induced diabetic RAG1-deficient and control diabetic mice, suggesting that the major mechanism for the inflammatory sequence was not affected by the absence of lymphocytes in their model (Lim et al., 2010); however, these results must be confirmed in additional studies, such as those using much stronger diabetic kidney fibrosis models (Sugimoto et al., 2007), to determine whether this observation is generalizable in diabetic nephropathy. It is not clear how or whether the various inflammatory response processes can affect disease-specific responses and subsequent tubule-interstitial injury from fibrosis.

Evidence from renal biopsies has shown that macrophage accumulation in diabetic kidneys predicts declining renal function (Duffield, 2010; Wang and Harris, 2011). STZ-induced diabetic animal models indicate that macrophage accumulation is associated with kidney fibrosis (Chow et al., 2004; Sugimoto et al., 2012). It is believed that macrophages play crucial roles in renal fibrogenesis (Duffield, 2010; Wang and Harris, 2011). Monocytes are recruited from circulating blood into the injured sites in response to tissue damage through cytokine-directed navigation, and subsequently recruited monocytes are differentiated into two broad but distinct subsets of macrophages: activated (M1) macrophages and alternatively activated (M2) macrophages (Ricardo et al., 2008; Lin et al., 2009). It is believed that M1 macrophages exhibit a typical pro-inflammatory phenotype via the generation of various chemokines, as well as ROS. M1 macrophages display pathogenic functions that lead to further tissue injury and fibrosis. In diabetic animal models, the depletion of the chemokines intercellular adhesion molecule-1 (ICAM-1) and monocyte chemoattractant protein-1 (MCP-1) diminishes macrophage accumulation and subsequent inflammation and tissue damage (Chow et al., 2005, 2007b). Immunohistological analysis has revealed that macrophages accumulated in diabetic kidney injury sites exhibit inducible nitric oxide release, CD169, and phosphorylated p38 mitogen-activated protein kinases (Adhikary et al., 2004; Chow et al., 2007a,b). Macrophage scavenger receptor-A-deficient mice are protected from interstitial fibrosis in diabetic nephropathy associated with the amelioration of microinflammation (Usui et al., 2007). The depletion of macrophages has been shown to protect and restore renal fibrosis after various injuries in non-diabetic animal models. The reconstitution of macrophages can worsen existing fibrotic lesions, thereby demonstrating that they have a profibrotic role in renal fibrogenesis (Henderson et al., 2008; Ko et al., 2008a; Lin et al., 2009). Macrophage activation status is also a major determinant of their pro-fibrogenic ability. The infusion of toll-like receptor 9 (TLR9) agonist-activated macrophages exaggerated disease progression in doxorubicin-induced nephropathy in mice, whereas resting macrophages did not induce disease progression (Wang et al., 2008).

Dendritic cells originate from the same bone marrow myeloid progenitor cells as macrophages, and they abundantly accumulate in normal kidney interstitium (John and Nelson, 2007; Kaissling and Le Hir, 2008; Teteris et al., 2011). DCs have important roles in the regulation of immune tolerance and the mounting of robust immune responses to pathogens. Macconi et al. (2009) reported that DCs can produce antigenic peptides from albumin through a proteasome-dependent pathway in a remnant kidney model. Subsequently, such peptides activate CD8^+^ T cells, suggesting that in proteinuric diseases, renal DCs capture and carry filtered antigens to T cells, leading to the production of pro-inflammatory cytokines. The crucial roles of DCs in renal disease progression and fibrogenesis have been demonstrated in DC-depleted mice, which exhibit renal protection via the overexpression of the model antigens, ovalbumin and hen egg lysozyme, in glomerular oocytes and nephrotoxic serum nephritis models (Heymann et al., 2009; Hochheiser et al., 2011a). Interestingly, later nephrotoxic nephritis (NTN) models revealed that DC depletion in late-stage nephritis halted disease progression but that early-stage depletion augmented disease progression, suggesting that DCs have a disease phase-specific role in certain disease models (Hochheiser et al., 2011a,b). The significance of DCs in diabetic kidney fibrosis remains unclear (Tu et al., 2010).

The role of mast cells in renal fibrogenesis remains controversial and unclear (Kanamaru et al., 2006; Timoshanko et al., 2006; Holdsworth and Summers, 2008). In diabetic kidney disease, mast cell numbers have been correlated with interstitial fibrosis (Hiromura et al., 1998; Sakamoto-Ihara et al., 2007). However, analyses of mast cell-deficient experimental animal models have suggested that mast cells have renal-protective roles. Indeed, in experiments, mast cell-deficient mice exhibited increased mortality and histopathological deteriorations in anti-GBM syndrome disease models (Kanamaru et al., 2006). Interstitial fibrosis was augmented in mast cell-deficient mice compared to control animals, which was demonstrated using a puromycin amino nucleoside-nephritis model (Miyazawa et al., 2004).

The evidence described above clearly demonstrates that the activation of inflammatory lymphocytes, macrophages, and DCs is essential for fibrotic kidney disease initiation and progression, including diabetic kidney disease.

### INFLAMMATION: FIBROBLAST ACTIVATION

The local accumulation of profibrotic cytokines in the microenvironment following kidney injury leads to the activation of ECM-producing cells, which are essential for renal fibrogenesis. As discussed above, almost all cell types in the tubulointerstitium of the kidneys, such as residential fibroblasts, tubular epithelial cells, vascular smooth muscle cells, and a subset of macrophages, are responsible for producing ECM. The fundamental matrix-producing cells that generate a large amount of interstitial matrix components, including fibronectin and type I and type III collagens, are fibroblasts (Strutz and Zeisberg, 2006). Profibrotic cytokine transforming growth factor-beta (TGF-βs) has been shown to play an essential role in this process, and the inhibition of TGF-βs or the TGF-β-stimulated smad transcriptional factor signaling pathway blockade has been shown to exhibit anti-fibrotic effects (Border and Noble, 1994; Miyazono, 2000; Kanasaki et al., 2003, 2011; RamachandraRao et al., 2009; Takakuta et al., 2010; Hills and Squires, 2011; Lan, 2011; Sharma et al., 2011; Choi et al., 2012).

TGF-βs modulate the overall response by affecting different receptors and downstream signaling. Active TGF-βs have profibrotic effects, and latent TGF-βs have an anti-fibrotic effect (Lan, 2011; Meng et al., 2013). The smad pathway is a downstream signaling pathway that can be influenced by non-TGF-βs molecules, such as angiotensinogen and advanced glycation end products. Among the smad molecules, smad 2 and smad 7 are likely renoprotective, and smad 3 is pathogenic (Meng et al., 2010, 2013; Lan, 2011; Lan and Chung, 2012).

In diseased kidneys, activated fibroblasts express α smooth muscle actin (αSMA) and are often referred to as myofibroblasts. These types of cells possess unique contractile properties (Strutz and Zeisberg, 2006). In the renal fibrosis process, renal myofibroblasts are believed to be an activated fibroblast phenotype that essentially contributes to ECM production and deposition in tubulointerstitial fibrosis (Strutz and Zeisberg, 2006). Several studies have examined the origin, activation, and regulation of these matrix-producing myofibroblasts (Grande and Lopez-Novoa, 2009; Meran and Steadman, 2011).

**FIGURE 1:**
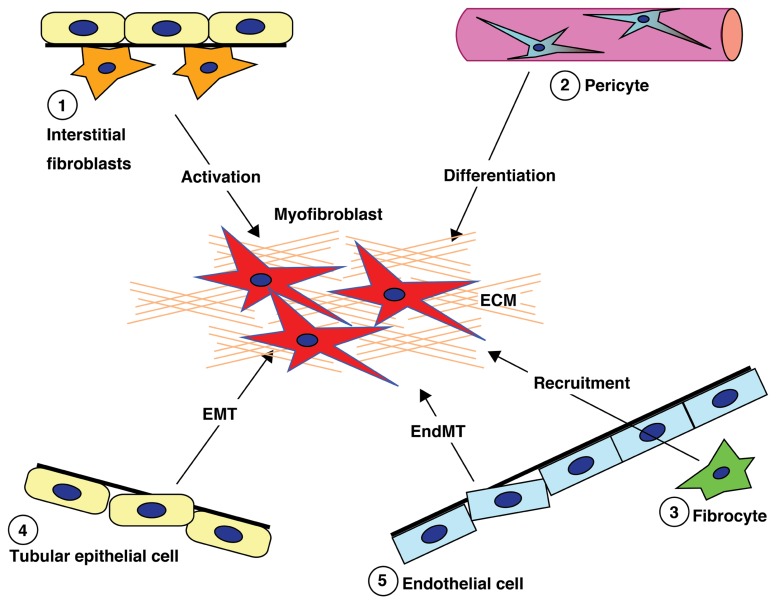
**Diverse origins of fibroblasts**. Kidney fibrosis is a well-coordinated event originating from various sources (1, interstitial cells; 2, pericytes; 3, fibrocytes; 4, tubular epithelial cells; 5, endothelial cells) and processes (recruitment, activation, differentiation, epithelial-to-mesenchymal transition, and endothelial-to-mesenchymal transition) that contribute to myofibroblast activation/formation.

**Figure 1** briefly summarizes the five well-reported sources of matrix-producing myofibroblasts, including the activation of residential fibroblasts, differentiation of pericytes, recruitment of circulating fibrocytes, and conversion of tubular epithelial cells and endothelial cells into mesenchymal cells (Barnes and Gorin, 2011). The relative contribution to and even the existence of each particular myofibroblast-generating pathway in renal fibrosis is still a topic of intense debate (Zeisberg and Duffield, 2010). Difficulty with identifying and tracking the lineage of fibroblasts, matrix-producing myofibroblasts, and other cell types due to the lack of specific markers has made the theory very controversial. Despite this controversy, there is no doubt that such matrix-producing fibroblasts exhibit significant heterogeneity.

### INFLAMMATION: ACTIVATION OF RESIDENTIAL FIBROBLAST AND PERICYTES

**FIGURE 2:**
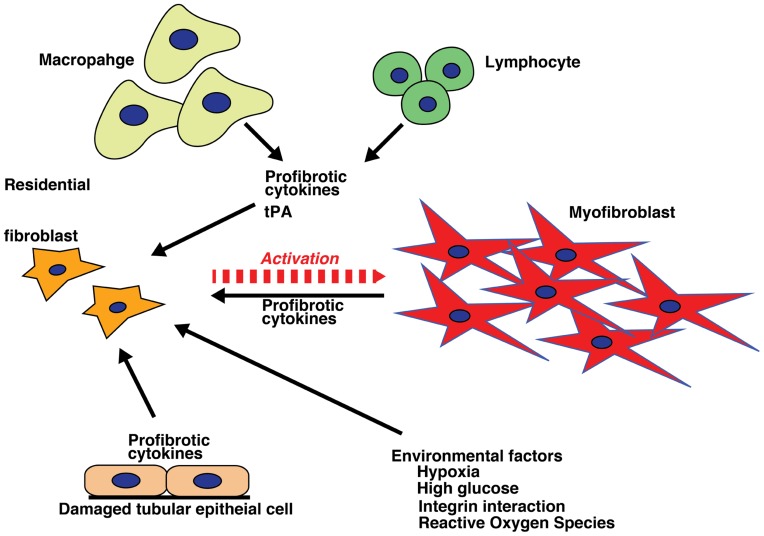
**Activation of fibroblasts**. Internal milieu changes (hypoxia, high glucose, integrin interactions, and production of reactive oxygen species) and profibrotic inflammatory cytokines from varied cell sources (macrophages, lymphocytes, and damaged tubular epithelial cells) result in fibroblast activation, which initiates and sustains the process of fibrosis.

Historically, all matrix-producing myofibroblasts were thought to originate from residential fibroblasts by phenotypic activation following renal injury and inflammation (Hewitson, 2009; **Figure 2**). This concept has recently been debated and challenged (Strutz and Zeisberg, 2006; Grande and Lopez-Novoa, 2009; Zeisberg and Duffield, 2010).

Fibroblasts are localized in the interstitial space between the capillaries and the epithelia in normal kidneys. They are organized as a network spread throughout the renal parenchyma, where they stabilize and organize the tissue architecture (Kaissling and Le Hir, 2008). Morphological analysis has revealed that these fibroblasts are stellate shaped and display a rough endoplasmic reticulum, collagen-containing granules and actin filaments (Strutz and Zeisberg, 2006). They exhibit multiple cell processes, which enable them to interact with the tubular and capillary basement membranes (Kaissling and Le Hir, 2008). In quiescent states, interstitial fibroblasts express CD73 (also known as ecto-5′-nucleotidase) in their plasma membrane and are responsible for producing erythropoietin (Kaissling and Le Hir, 2008; Paliege et al., 2010). These cells also express platelet-derived growth factor receptor β (PDGFRβ; Floege et al., 2008) and fibroblast-specific protein 1 (FSP1; also known as S100A4), a small protein that exhibits a calcium-binding motif and is associated with the cytoskeleton (Wynn, 2007). Under normal conditions, fibroblasts are responsible for maintaining the homeostasis of the interstitial matrix against physiologic conditions by producing an essential, basal level of ECM components. Following profibrotic cytokine inflammation and mechanical stress, these interstitial fibroblasts undergo phenotypic change into myofibroblasts by expressing αSMA, and they begin to produce a large amount of ECM components. Myofibroblasts can express both FSP1 and PDGFRβ. In addition, myofibroblasts can also express *de novo* vimentin (an intermediate filament protein). The myofibroblast phenotype is indeed a mixture of both matrix component-producing activated fibroblasts and αSMA-expressing highly contractile smooth muscle cells.

Fibroblasts become activated by stimulation with cytokines, including TGF-βs, connective tissue growth factor (CTGF), platelet-derived growth factor (PDGF), and fibroblast growth factor 2 (FGF2; Liu, 2011). In addition to cytokine-mediating reactions, direct cell–cell interactions (with leukocytes and macrophages), ECM–integrin interactions, hypoxia, and hyperglycemia could contribute to the activation process (Chow et al., 2004, 2007a; Liu, 2011). In tubulointerstitial fibrosis, renal fibroblasts maintain an activated status, even after the initial injury and insult has ceased (Liu, 2011). Activated fibroblasts are characterized by two key features: proliferation and myofibroblastic activation (Strutz and Zeisberg, 2006). However, the proliferation of residential fibroblasts is also controversial, and some reports have indicated that no proliferation occurs in unilateral ureteral obstruction renal fibrosis models (Yamashita et al., 2005). Myofibroblastic activation is illustrated by αSMA expression and matrix production (Strutz and Zeisberg, 2006). Both fibroblasts and myofibroblasts have the ability to proliferate when stimulated with cytokines. This fibroblast proliferation results in the expansion of the fibroblast population and ECM deposition in the interstitial space in damaged kidneys (Strutz and Zeisberg, 2006). Fibroblasts are activated and proliferate in response to several cytokines and mitogens, such as PDGF, TGF-β, basic FGF2, and CTGF. These cytokines are also potentially derived from activated macrophages (Strutz et al., 2000; Bottinger, 2007; Hewitson, 2009; Phanish et al., 2010; Boor and Floege, 2011; Ostendorf et al., 2012).

Tissue-type plasminogen activator (tPA), which can also be derived from activated macrophages, is another critical factor in kidney fibrosis (Hu et al., 2007, 2008; Hao et al., 2010; Lin et al., 2010). tPA plays crucial roles in the pathogenesis of renal interstitial fibrosis through a variety of mechanisms, such as protection from apoptosis and induced mitogenesis (Hu et al., 2007, 2008; Hao et al., 2010; Lin et al., 2010). In renal interstitial fibroblasts, tPA also induces the expression of matrix metalloproteinase-9 (MMP-9), which causes destruction of the tubular basement membrane (TBM), subsequently leading to tubular EMT (Hu et al., 2006). tPA also acts independently of its protease activity and induces myofibroblast activation of quiescent interstitial fibroblasts through low density lipoprotein (LDL) receptor-related protein 1 (LRP-1)-mediated recruitment of β1 integrin signaling in rat kidney fibroblasts (Hu et al., 2006, 2007). Two downstream effectors of integrin signaling, focal adhesion kinase and integrin-linked kinase (ILK), control fibroblast proliferation and matrix production, respectively (Hao et al., 2010). tPA also acts as a survival factor that protects renal interstitial fibroblasts/myofibroblasts from apoptosis, resulting in the expansion of the myofibroblast population in diseased kidneys.

Some studies have suggested that vascular pericytes are a major source of myofibroblasts in fibrotic kidneys (Lin et al., 2008; Humphreys et al., 2010; Schrimpf and Duffield, 2011). However, this concept remains controversial (Zeisberg and Duffield, 2010), largely due to difficulties in defining what constitutes a pericyte. Pericytes are one subset of the stromal cells, which offer endothelial partial support by covering capillary walls to aid in stabilization. The markers for pericytes used in several reports that indicate pericyte–myofibroblast conversions, such as PDGFRβ, are not absolutely specific and are indeed expressed in many cell types, including fibroblasts. Fibroblasts are intricately connected to the capillaries via cell processes in the renal interstitium (Liu, 2011). Some reports have suggested that once kidneys are damaged, pericytes no longer adhere to the endothelium and that thereafter, these cells migrate, proliferate, and finally differentiate into myofibroblasts (**Figure 2**; Lin et al., 2008; Humphreys et al., 2010). In addition, the blockade of PDGFR signaling by imatinib reduces the number of myofibroblasts and restores renal damage in UUO models (Chen et al., 2011). These results also do not entirely support pericyte conversion into myofibroblasts but do indicate that PDGF signaling is a crucial player in kidney fibrogenesis (LeBleu and Kalluri, 2011). Although the pericyte conversion concept is interesting, it requires further study to verify whether pericytes and interstitial fibroblasts are, in fact, the same entity (Kaissling and Le Hir, 2008).

### INFLAMMATION: BONE MARROW-DERIVED CELL RECRUITMENT

Fibrocytes, which are a subset of bone marrow-derived circulating monocytes, also display fibroblast-like features in the peripheral blood and are another possible source of fibroblasts (Herzog and Bucala, 2010). Human fibrocyte precursors can be co-purified together with CD14^+^ monocytes (Peng et al., 2011). In mice, the fibrocyte transition from monocytes is enhanced with the expression of CD11b, CD115, and Gr1 (Niedermeier et al., 2009). The transition is stimulated by direct contact with activated CD4+ lymphocytes and is mediated via a mammalian target of rapamycin (mTOR)–PI3-dependent pathway (Niedermeier et al., 2009). Differentiated fibrocytes are negatively regulated by the Fcγ receptors CD64 and CD32, as indicated by the inhibition of such receptors with serum amyloid P, which inhibits fibrocyte accumulation in human and experimental animal models (Pilling et al., 2003, 2006, 2007) through immunoreceptor tyrosine-based inhibitory motif-dependent mechanisms (Pilling et al., 2006). Fibrocyte differentiation is stimulated by the Th2 cytokines interleukin (IL)-4 and IL-13 and TGF-β1, which is aided by integrin β1 and inhibited by Th1 cytokines such as interferon-gamma (IFN-γ), tumor necrosis factors (TNF), and IL-12. TLR2 agonists indirectly inhibit fibrocyte differentiation, and it is likely that this inhibition involves the mechanisms by which other cell types in the peripheral blood mononuclear cell population secrete unknown fibrocyte differentiation inhibitory factors (Maharjan et al., 2010). As TLR2 is a receptor for immunopathogens, it is possible that some bacterial signals can inhibit fibrocyte differentiation and may thus slow wound closure (Maharjan et al., 2010).

Fibrocytes display the characteristics of both fibroblasts and hematopoietic cells, as they are spindle-shaped and express the hematopoietic cell marker CD45. Fibrocytes have the ability to produce type I collagen (Pilling et al., 2009; Wada et al., 2011). Interestingly, fibrocytes also display certain chemokine receptors, such as CCR1, CCR2, CCR7, CXCR4 in mice (Sakai et al., 2006; Mehrad et al., 2009; Ekert et al., 2011; Scholten et al., 2011) and CCr2, CCR3, CCr5, and CXCR4, as well as the β1 integrin subunit, and semaphorin 7a in humans (Abe et al., 2001; Mehrad et al., 2009; Ekert et al., 2011; Gan et al., 2011). Following kidney injury, fibrocytes have been hypothesized to mobilize, infiltrate the renal parenchyma and participate in fibrogenesis. The differentiation of fibrocytes is also regulated through the other inflammatory cells, such as CD4^+^ T cells, via the production of cytokines (Niedermeier et al., 2009). The profibrotic inflammatory cytokines IL-4 and IL-13 induce the differentiation of fibrocytes, whereas anti-fibrotic cytokines such as IFN-γ and IL-12 inhibit fibrocyte differentiation, as expected (Shao et al., 2008). Interestingly, the calcineurin inhibitor cyclosporine promotes fibrocyte differentiation, suggesting a possible explanation for cyclosporine-induced nephrotoxicity in clinical settings (Niedermeier et al., 2009). The inhibition of angiotensin II type-1 receptor signaling prevents the accumulation of fibrocytes in the kidneys as well as the bone marrow in mouse models of renal fibrosis (Sakai et al., 2008).

Nevertheless, the significance of fibrocytes in renal fibrogenesis remains controversial. Similar to the issues with other theories, there are no specific markers for these cells to allow a clear distinction of fibrocytes from other types of cells, such as monocytes, macrophages, fibroblasts, and myofibroblasts. Adding to the controversy, it has been shown that there are subpopulations of fibrocytes (Pilling et al., 2009). The role of fibrocytes, or bone marrow-derived cells, in renal fibrosis is inconsistent, yet (Iwano et al., 2002; Roufosse et al., 2006; Lin et al., 2008; Niedermeier et al., 2009) studies have shown that a considerable ratio of all collagen-producing fibroblasts in a mouse model of UUO that originate from fibrocytes or bone marrow-derived cells (Iwano et al., 2002; Niedermeier et al., 2009); however, other studies using the same model have reported contradictory results (Roufosse et al., 2006; Lin et al., 2008, 2009). If there is a specific fibrocyte lineage, it must contribute to the tissue repair process. The role of bone marrow cells in tissue repair has been reported in studies of genetic defects in type IV collagen α3 chain knockout mice, the model of human Alport syndrome (Sugimoto et al., 2006). Therefore, some bone marrow cell lineages must be involved in tissue repair. As fibrosis is the end result of uncontrolled tissue repair and wound healing processes, it is reasonable that fibrocytes have a role in fibrogenesis when they accumulate excessively at the injured site.

### INFLAMMATION: EMT AND EndMT

During embryogenesis, the epithelia exhibit high plasticity, and they can alternate between epithelia and mesenchyme through the processes of EMT and mesenchymal-to-epithelial transition (MET; Thiery, 2002; Kalluri and Neilson, 2003). When organ development is completed, the epithelia typically display specialized functions, and this specialization is believed to be a terminal differentiation (Gumbiner, 1992; Yeaman et al., 1999). However, recent biological evidence has shed new light on the plasticity of epithelia, which were formerly considered terminally differentiated cells. Using human renal biopsy samples, the number of tubular epithelial cells with EMT features has been shown to be associated with serum creatinine and the degree of damage (Rastaldi et al., 2002). In addition, EMT has been reported in canine glomerulonephritis (Aresu et al., 2007). More than 100 studies have demonstrated the potential significance of EMT in kidney fibrosis by examining the phenotypic conversion of tubular cells in animal models as well as in renal biopsy samples from various kidney disease patients (Yang and Liu, 2002; Zeisberg et al., 2003; Hertig et al., 2008; Boonla et al., 2011; Togawa et al., 2011). Morphological evidence indicates that epithelial cells do indeed traverse the disrupted TBM into the interstitium after kidney injury in UUO in mice (Yang et al., 2002). EMT in human diseases, such as inflammatory bowel diseases and several cancers, has been reported (Kalluri and Weinberg, 2009). This evidence strongly suggests that epithelial plasticity and the occurrence of EMT play a role in certain human disease conditions.

**FIGURE 3:**
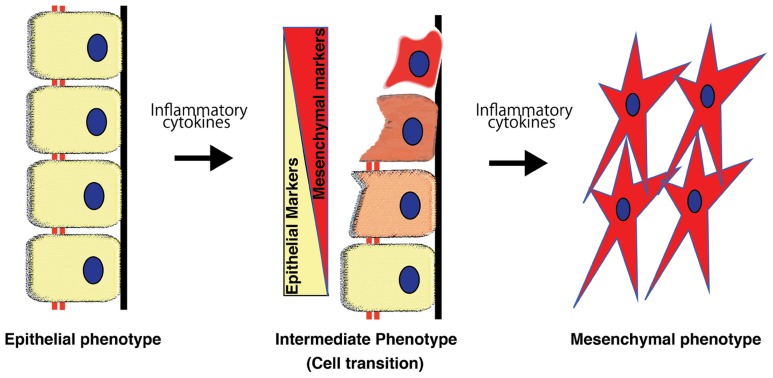
**Process of epithelial-to-mesenchymal transition**. Epithelial-to-mesenchymal transition is a phenomenon that is well-orchestrated by inflammatory cytokines and results in epithelial cells gradually losing their cell markers and acquiring the mesenchymal phenotype.

Using a genetic-lineage tracking, Iwano et al. (2002) reported that over one-third of FSP1^+^ interstitial fibroblasts originated from the tubular epithelia in a mouse model of obstructive nephropathy. Although such evidence strongly suggests the presence of EMT in tissue fibrosis, some recent experimental studies using lineage-tracing have reported that the epithelial or endothelial origin of fibroblasts is unclear (Humphreys et al., 2010; Li et al., 2010). These reports were questioned in a review (Zeisberg and Duffield, 2010). EMT-associated matrix-producing mesenchymal cells definitely exist, but EMT in fibrogenesis does not necessarily indicate de novo production of fibroblast from tubular epithelial cells (**Figure 3**), which is the extreme outcome of EMT (Kalluri and Weinberg, 2009; Zeisberg and Duffield, 2010; **Figure 3**). The number of epithelial cells undergoing EMT does not necessarily need to be identical or even similar to the number of cells that become fibroblasts (Zeisberg and Duffield, 2010). Fibroblasts proliferate at the injured site regardless of their origin. Therefore, even if the original EMT-derived fibroblasts were a minor population to begin with, they would proliferate and constitute a large portion of the fibroblasts, producing huge amounts of ECM components (Kalluri and Weinberg, 2009; Zeisberg and Duffield, 2010). Furthermore, as described above, EMT is a dynamic and even reversible process, and thus, intermediate EMT in tubular epithelial cells is more abundant compared to the extreme fibroblast end points of EMT. Therefore, the ratio of epithelial cells proceeding to complete EMT and complete conversion into fibroblasts is reasonably very low and depends heavily on disease model factors. It is likely that factors such as longer exposure to the micro-inflammatory milieu and the persistence of raised cytokine pressure are more likely to induce EMT (Liu, 2004; Kalluri and Weinberg, 2009; Zeisberg and Duffield, 2010).

Epithelial-to-mesenchymal transition is an important and useful therapy target in treating kidney disease. The blockade of EMT with various agents, such as bone morphogenetic protein (Zeisberg et al., 2003; Sugimoto et al., 2007), hepatocyte growth factor (Yang and Liu, 2002), ILK inhibitor (Li et al., 2009b), Wnt antagonists (Surendran et al., 2005; He et al., 2009), and paricalcitol (Tan et al., 2006), as well as the induction of endogenous heat shock protein (Wynn, 2007; Zhou et al., 2010), has been shown to ameliorate renal fibrosis and preserve kidney function in various animal models. Nuclear factor (erythroid-derived 2)-like 2 (Nrf2), a master regulator of oxidative stress and a potential drug target for diabetic nephropathy (Pergola et al., 2011; Li et al., 2012), has an inhibitory effect on the EMT process via interaction with heme oxygenase-1 (Shin et al., 2010). In addition, it was recently shown that BMP7 receptor Alk3 in renal tubules is essential for anti-fibrogenesis and tissue repair in the kidneys; Alk3-agonistic compounds were also shown to exhibit renal protection in experimental kidney fibrosis models, including models of diabetic nephropathy; this renal protection was associated with the inhibition of EMT, inflammation, and apoptosis (Sugimoto et al., 2012).

Fibroblasts and/or myofibroblasts can be derived from capillary endothelium through the EndMT (Zeisberg et al., 2008; Li et al., 2009a). EndMT is considered to be a unique form of EMT, as endothelial cells are a specialized type of epithelia. Zeisberg et al. (2008) investigated the contribution of EndMT in renal fibrosis in three mouse models of chronic kidney disease, including STZ-induced diabetic nephropathy. When analyzed according to the presence of endothelial marker CD31 and the (myo)fibroblast markers αSMA and FSP1 in conjunction with lineage tracing, approximately 40% of all FSP-1-positive and 50% of αSMA-positive cells in STZ kidneys were also CD31 positive (Zeisberg et al., 2008), suggesting a significant contribution of EndMT in kidney fibrosis. Li et al. (2009a) also reported a contribution of EndMT in diabetic glomerular sclerosis as well as interstitial fibrosis in early diabetic mice.

Biological analysis has revealed that EndMT is also regulated by a combination of various inflammatory cytokine pressures. Although the role of inflammatory cytokines in EndMT and kidney fibrosis has not been clearly established, it is likely that inflammation is a critical mediator in EndMT. In an inflammatory bowel disease model, Rieder et al. (2011) clearly demonstrated that either a combination of TGF-β1, IL-1β, and TNF-α or activated lamina propria mononuclear cell supernatants induced morphological and phenotypic changes consistent with EndMT; each inflammatory cytokine alone had limited effects on endothelial phenotypic changes (Rieder et al., 2011). Another recent report indicated that the inflammatory transcriptional factor nuclear factor-kappaB (NFκB) determined inflammation-induced EndMT (Maleszewska et al., 2012). These reports suggest that inflammation has a crucial role in the pathogenesis of EndMT. TGF-βs, IL-1β, and TNF-α expression are indeed increased at the site of kidney injury in diabetes (Lim and Tesch, 2012). It has been shown that IL-1, IL-6, IL-18, and TNF-α are likely relevant in diabetic nephropathy.

Fibroblasts heterogeneity has been established, and such heterogeneity could be explained in part by various fibroblast origins, such as residential fibroblast activation, pericyte conversion, fibrocyte origination, and EMT/EndMT. In addition, kidney fibroblasts in diabetic kidneys could be somewhat different from those of other kidney diseases. Fibroblasts isolated from diabetic skin ulcers exhibited diverse morphological and functional characteristics when compared to normal skin fibroblasts (Loots et al., 1999; Xu et al., 2012b). It also likely that fibroblast activation from fibroblasts, pericytes, or fibrocytes is an early event (Kaissling and Le Hir, 2008), whereas EMT and/or EndMT contribute at a late stage after longer, sustained injury (**Figure 2**; Liu, 2004). In each type of fibroblast activation process and the sequential process of kidney fibrogenesis, inflammatory cells and their produced cytokines play a crucial role.

### VARIATION IN SUSCEPTIBILITY FOR DIABETIC NEPHROPATHY: THE ROLE OF VARIATION IN INFLAMMATORY RESPONSE

Variations in conventional risk factors, which include variations in blood sugar control, blood pressure control, and proteinuria control, can contribute to variations in diabetic nephropathy susceptibility (Fernández Fernández et al., 2012).

Genetic polymorphisms in a variety of molecules involved in pathogenic pathways of diabetic nephropathy could contribute to the variation in individual susceptibility (Brorsson and Pociot, 2011). Genetic polymorphisms can affect important pathogenic pathways such as the renin–angiotensin pathway, the inflammatory cytokine pathway, the nitric oxide pathway, the bradykinin pathway, the Wnt pathway, Notch signaling, and the matrix metalloprotease-related pathway, and have an impact on the development and progression of diabetic nephropathy (Maeda, 2008; Brorsson and Pociot, 2011; Kure et al., 2011).

The cytokine milieu is more important than individual cytokine levels because cytokines exhibit pleiotropism, redundancy, synergism, antagonism, *trans*-modulation, and *trans*-signaling. The effect of the overall cytokine milieu, rather than individual cytokine levels, influences this effect (Navarro-Gonzalez et al., 2011; Elmarakby and Sullivan, 2012). The variations in the cytokine response that is mediated by genetic variation can contribute to the variations observed in diabetic nephropathy.

Variations in TLRs also modulate the response based on the level of antigenic exposure and their location in particular organs (Huebener and Schwabe, 2012). Too much immunity results in autoimmune disorders, and too little immunity results in infections. Thus, optimal immunity requires a balance between autoimmunity and opportunistic infections (Thomas, 2010; Fujio et al., 2012). There are no exact cutoff values that define high, optimal, or low immunity levels for inflammatory mediators. Acute and transient elevations in cytokines lead to different activity than does chronic low-grade elevation. Infection and injury that produce transient stimulus of the immune or inflammatory systems resolve without resulting in fibrosis (Serhan et al., 2007). If an infection or injury is chronic (e.g., tuberculosis or non-healing wounds), fibrosis can result (dos Santos et al., 2012; Franchi et al., 2012).

Persistent injury or inflammation after an unknown period of time can result in permanent changes in the inflammatory response, resulting in the unregulated production of profibrotic factors. Even controlling the inciting stimulus at that time may not be able to prevent the progression of the disease due to epigenetic alterations (Gaede et al., 2008; Bechtel et al., 2010; Xu et al., 2012a).

A multifaceted and sequential approach may be needed for the management of diabetic kidney fibrosis (Gaede et al., 2008). Diabetic kidney disease pathways are redundant with respect to the multiple pathogenic factors that can activate the final fibrosis pathway. At different stages of disease, different pathogenic pathways predominate, necessitating the initiation of therapies in a sequential manner.

Glycemic control is beneficial for preventing micro-vascular complications in newly diagnosed diabetic patients, as reported by the DCCT and UKPDS trials, but it is not beneficial in established diabetic patients for the prevention of significant outcomes of diabetic nephropathy, as reported in recent trials (Hemmingsen et al., 2011; Coca et al., 2012; Slinin et al., 2012). Late-stage diabetes, hypertension, and proteinuria may be the predominant causes of the progression of diabetic nephropathy, necessitating antihypertensive/antiproteinuric measures to prevent further disease progression.

Multiple and sequential management includes the initial blood sugar control and blood pressure control, followed by anti-inflammatory, anti-EMT/EndMT, and anti-fibroblast activation medications at different stages of diabetic kidney disease (Goel and Perkins, 2012).

### PERSPECTIVE: SLOWING THE PROGRESSION OF DIABETIC KIDNEY DISEASE – THE ROLE OF INFLAMMATORY PATHWAYS

Existing therapies targeting glucose control, blood pressure control, and proteinuria reduction, alone or in combination, have failed to slow or reverse the progression of diabetic nephropathy. As is evident in the above discussion, the role of inflammation in the initiation and progression of diabetic nephropathy is undisputed.

Basic studies using various animal models have demonstrated pro-inflammatory cytokine gene activation in diabetic nephropathy (Navarro et al., 2005, 2006; Navarro-Gonzalez et al., 2009). Various existing drugs, which have anti-inflammatory activity, have been shown to slow or reverse diabetic kidney disease.

### ANTI-INFLAMMATORY THERAPIES

An aldosterone antagonist (spironolactone) inhibits NFκB, thereby inhibiting MCP-1 (Han et al., 2006). Pioglitazone, a peroxisome proliferator-activated receptor (PPAR) agonist, has been shown to slow diabetic nephropathy by downregulating various pro-inflammatory and profibrotic genes, such as NFkB, CCL2, TGF-β1, plasminogen activator inhibitor-1 (PAI-1), vascular endothelial growth factor (VEGF), etc. (Ko et al., 2008b). ACE inhibitors and ARBs suppress NFκB signaling and thereby suppress the inflammatory response (Kim et al., 2011). Pentoxifylline, TNF-α receptor fusion proteins and chimeric monoclonal antibodies have been shown to decrease TNF-α, a pro-inflammatory cytokine, thereby decreasing fibrosis (Navarro-Gonzalez et al., 2009). Bardoxolone mesylate is a novel triterpenoid agent with Nrf-2 agonistic activity that has been shown to have anti-inflammatory and anti-oxidant activity. It inhibits NFκB and Janus kinase/signal transducers and activators of transcription (JAK-STAT) signaling and acts as an anti-oxidant and inflammatory modulator (Zhang, 2013). This compound was expected to be a useful drug for diabetic nephropathy in an early clinical trial (Pergola et al., 2011); however, further clinical trials evaluating the renoprotective effects of this drug against diabetic nephropathy were terminated due to increased cardiac events in the drug-treated group (Zhang, 2013). Finally, cyclooxygenase (COX)-2, a pro-inflammatory enzyme responsible for the formation of inflammatory prostanoids, is induced in diabetic kidneys (Komers et al., 2001), and preclinical data suggest that COX-2 inhibitors decrease proteinuria and preserve glomerular structure in animal models of diabetic nephropathy (Cheng et al., 2002; Komers et al., 2007; Matsunaga et al., 2007; Nasrallah et al., 2009; Quilley et al., 2011). However, clinical trials have not yet revealed any significant clinical outcomes of COX-2 inhibition on diabetic nephropathy (Sinsakul et al., 2007; Cherney et al., 2008a,b). In addition, the enhanced expression of COX-2 is involved in maintaining adequate renal hemodynamics and function in some patients with diabetic nephropathy (Khan et al., 2001). Therefore, the inhibition of COX-2 would be harmful in a certain set of diabetic nephropathy patients.

In view of the current understanding of the central role of inflammation in diabetic nephropathy progression, future studies may focus on maintaining epithelial integrity, halting the process of EMT/EndMT transition and controlling the cytokine milieu.

Future diabetic nephropathy research could be directed toward inhibiting the inflammatory pathway in conjunction with conventional therapeutic strategies (Navarro and Mora, 2006; Rivero et al., 2009), although a few fundamental questions remain. Indeed, in contrast to the experimental animal model studies, anti-inflammatory therapy has been notably ineffective in halting kidney fibrogenesis in clinical settings. Furthermore, anti-inflammatory therapy has worsened clinical outcomes in patients with lung fibrosis and systemic sclerosis (Wynn, 2011; Murray et al., 2012). These reports suggest that anti-inflammatory therapy would not be effective or could even worsen clinical outcomes in patients with established diabetic nephropathy and fibrosis. Additionally, there is a possibility that inflammation is an important initiator of fibrosis, as described above, although in the advanced chronic fibroproliferative stage, inflammation would act in an alternative manner to facilitate organ repair and protection. Therefore, examining stage-specific inflammation in diabetic nephropathy and its associated fibrosis, as well as identifying reliable biomarkers, is required before inflammation can be used as a therapeutic target in the treatment of this condition.

## CONCLUSION

Diabetic nephropathy is a devastating kidney disease that contributes to the majority of end-stage kidney disease cases worldwide, and this condition is associated with a higher frequency of cardiovascular events. Diabetic nephropathy is associated with the activation of a variety of pathways that lead to the progression of kidney disease. Among these pathways, inflammatory pathway activation plays a central role. The inflammatory pathway leads to the activation and recruitment of the fibroblasts, which in turn initiate and sustain the fibrotic process. Apart from conventional glucose control and blood pressure control, targeting the specific pathways that lead to the activation of inflammation and fibroblasts could be a new and effective intervention in the management of diabetic nephropathy.

## Conflict of Interest Statement

The authors declare that the research was conducted in the absence of any commercial or financial relationships that could be construed as a potential conflict of interest.
